# D6/ACKR2

**DOI:** 10.3389/fimmu.2015.00280

**Published:** 2015-06-05

**Authors:** Gerard J. Graham

**Affiliations:** ^1^University of Glasgow, Glasgow, UK

**Keywords:** chemokines, receptors, cell surface, inflammation, cancer, placenta

At the outset, it is worth noting that, for historical reasons, I have referred to D6/ACKR2 as “D6” throughout the majority of this essay. Issues relating to the complexity of D6 nomenclature are discussed below.

## In the Beginning …

Very much in keeping with the name of the chemokine receptor subfamily to which D6 belongs, the majority of my research in the field of chemokine biology has been “atypical” in the sense that it has rarely focused on classical immunological roles for these molecules. Indeed, the story behind the discovery of D6 starts from an unusual research perspective! In 1988, I was employed as a postdoctoral researcher in the laboratory of Prof. Ian Pragnell who became a close friend, and with whom I enjoyed many international adventures. Ian, at the time, was interested in trying to identify inhibitors of hematopoietic stem cell proliferation with the idea that these might be used as myelo protective agents during cancer chemotherapy. He had identified an “activity” in the conditioned media of J774 cells, which was capable of inducing quiescence in primary murine and human hematopoietic stem cells and my job was to purify and characterizes this factor. The protein responsible for this activity proved reasonably easy to purify and turned out to be CCL3 although, at the time, we called it stem cell inhibitor, or SCI. This work was published in Nature in 1990 (1) and represented the first demonstration of a role for chemokines in regulating stem cell function and this, of course, has now become a prominent sub specialty in the chemokine field. The next objective was to clone the receptor for this stem cell inhibitor. I spent a frustrating period of time trying to “expression clone” this CCL3 receptor from “stem cell like” cell lines but these approaches met with little success. Alternative approaches were needed.

## The Cloning of D6

In 1993, I was lucky to be able to recruit, to the group, a very talented young post doctoral researcher named Rob Nibbs who was (and is!) a highly gifted molecular biologist. Rob then set about developing new strategies for the cloning of CCL3 receptors. At this stage, only CCR1 had been identified and we had shown that this was not involved in mediating the stem cell inhibitory effects of CCL3 suggesting that an, as yet unidentified receptor, was key. Rob set his mind on using a degenerate genomic PCR cloning strategy based on the emerging indications that the majority of the coding regions for chemokine receptors were incorporated within a single genomic exon. This strategy led to the identification of a number of novel murine chemokine receptors. Frustratingly, as is the way in competitive science, a number of these receptors were published by other groups just as we were drafting out our publications reporting their cloning. However, one receptor that we had identified was not published by other groups and was reported by us under the name of “D6” in 1997 (2). Notably, Steiner and colleagues also reported the cloning of D6 around the same time (3) but they did not pursue further biological studies of this molecule. Shortly after the cloning of murine D6, we reported the cloning of the human homolog (4). One of the curious features of both murine and human D6 was that they lacked the canonical DRYLAIV motif, which had been found in all other cloned chemokine receptors and which was regarded as being important for cellular signaling. This suggested an unusual aspect to the biology of D6 function.

## The Expression of D6

In collaboration with Paul Ponath, and his colleagues at Leukosite (a former Biotech company in the United States), we generated monoclonal antibodies to human D6 and used these to demonstrate that the predominant cells expressing D6 in adult tissues were lymphatic endothelial cells (5). In addition, strong D6 expression was seen throughout the syncytiotrophoblast layer in the placenta and expression was also noted on some leukocyte subtype (6). Therefore, again in keeping with the atypical nature of this molecule, D6 expression patterns were markedly different from the other chemokine receptors further suggesting unusual aspects to D6 biology.

## Insights into D6 Function

Exhaustive ligand binding studies demonstrated that D6 was a highly promiscuous receptor capable of binding the majority of (if not all) inflammatory CC-chemokines. It did not bind homeostatic CC-chemokines not did it bind CXC, XC, or CX3C chemokines. We therefore characterized it as a promiscuous receptor with a specificity for inflammatory CC-chemokines. Binding affinities for the ligands were generally in the high pM and low nM range and therefore equivalent to those seen with the other chemokine receptors. In keeping with the altered DRYLAIV motif, and contrary to data reported in our initial cloning paper (2) (which we presume was a consequence of a mutation introduced into the receptor clone used), we were never able to demonstrate signaling through D6 or chemotactic responses in cells expressing this receptor. This led us to the tentative assumption that D6 was a non-signaling chemokine receptor [more recent observations from our Milan colleagues have suggested “atypical” signaling pathways downstream of ligand binding by D6 (7)] but quite what this meant for function was not immediately apparent.

The breakthrough was provided when Alberto Mantovani and Massimo Locati and their group in Milan demonstrated that D6 was capable of internalizing and effectively scavenging its ligands (8). Shortly after, we showed that D6 spontaneously internalized and recycled to the cell membrane in any cell type, which it was expressed (9). Together, these observations led to a model of D6 function, which proposed that D6 does not support cellular migration but that, following binding, it internalizes ligand and deposits it in lysosomes for intracellular degradation. The great advantage of D6 is its promiscuity and all the analyses that we, and our Milan colleagues, have performed have demonstrated that it is an exquisitely efficient scavenger of inflammatory CC-chemokines. Notably, all these data were generated using *in vitro* approaches and so the next challenge was to demonstrate a role for D6 *in vivo* and to see if such a role was compatible with *in vivo* chemokine scavenging activity.

## D6 *In vivo*

Our next target was to generate D6-deficient mice to allow us to study their responses in a range of inflammatory models. At the time, this was not an area of expertise that we possessed and so I initiated a collaboration with our friends Don Cook and Sergio Lira who were expert in this area and who were both, at the time, employed by the Schering-Plough Research Institute in Kenilworth New Jersey. Don quickly generated the D6-deficient mice and sent them to us for analysis. Import of these mice into Scotland, however, did not go quite as smoothly as initially planned! During the flight from the United States to Scotland, the mice managed to gnaw through the wall of the container in which they had been kept. Once the authorities discovered this they were concerned that mice might have escaped into the electrics of the aeroplane and might therefore cause serious problems with the plane’s function. We therefore had to prove, without doubt, that no mice had escaped from the cage. Fortunately, the mice were not sufficiently interested in exploring the plane and we were able to demonstrate that all mice that had been sent remained in the cage. This was a massive relief as the cost of stripping down, and rebuilding, a Jumbo Jet to find a lost mouse would have bankrupted the Institute in which Rob and I were employed at the time! Anyway, the mice arrived safely and we proceeded to examine their responses in a relatively simple model of cutaneous inflammation involving the topical application of the phorbol ester TPA. What we found, and very much in keeping with a role for D6 as a scavenger of inflammatory chemokines, was that these mice displayed an inability to effectively resolve this cutaneous inflammatory response. Indeed, the mice developed a pathology that displayed remarkable similarities to human psoriasis. This work was published in Nature Immunology in 2005 (10) and was followed by numerous other studies in different tissue systems both from our own group and from the Milan group (11). Together these studies unequivocally demonstrated a role for D6 in the resolution of inflammatory response. The importance of D6 for scavenging inflammatory CC-chemokines was also reflected in other pathological phenotypes in D6-deficient mice. For example, and as mentioned above, a major site of D6 expression is the syncytiotrophoblast layer of the placenta and D6-deficient mice display enhanced susceptibility to miscarriage in response to maternal systemic inflammation (12). In addition, D6-deficient mice display exaggerated tumorigenic programs in a variety of inflammation-dependent cancer models. D6 is therefore a scavenger of inflammatory chemokines with important roles to play in a range of tissue and pathological, contexts. Notably, we have recently published evidence indicating a developmental role for D6 in regulating the density of lymphatic vessel networks in embryonic skin (13). Together these studies implicate the D6 in the regulation of pro-lymphangionenic macrophage proximity to developing lymphatic vessel networks and provide the first evidence of a role for inflammatory chemokines, and their regulators, in developmental processes.

## The Nomenclature Problem!

The name “D6” refers to nothing more complicated than the coordinates, on a multiwell plate, of the clone encoding this receptor. As mentioned above, we erroneously initially believed that D6 was a classical signaling molecule and therefore contacted the chemokine receptor nomenclature committee to register it. It was initially designated as CCR9. However, the Steiner group also requested a systematic nomenclature for their D6 clone around the same time and was provided with CCR10 as a designation. Therefore, for some time, this receptor was variously known as D6, CCR9, and CCR10! To confuse things even further the GenBank accepted name was “ccbp2” standing for chemokine binding protein-2. Eventually, both the CCR9 and CCR10 nomenclatures were assigned to other receptors and D6 became the accepted name for this molecule. However, most recently, we have developed a systematic nomenclature system for the entire atypical chemokine receptor family to which D6 belongs and refer to these as ACKRs. Within this IUPHAR approved nomenclature system D6 is now known as ACKR2, which is now its settled nomenclature (14).

## In Summary

Starting from an unusual standpoint, and with essential input and insights from our Milan colleagues, we have cloned and characterized D6/ACKR2 as a scavenger of inflammatory CC-chemokines and have demonstrated its importance for the resolution of inflammatory response in a variety of contexts. D6/ACKR2 provides a paradigm for the function of other members of the atypical chemokine receptor family and similarities with the function of ACKR3 and ACKR4 have already become apparent (15). We believe that this molecule has both diagnostic and therapeutic value although this potential has yet to be realized.

## Conflict of Interest Statement

The author declares that the research was conducted in the absence of any commercial or financial relationships that could be construed as a potential conflict of interest.
